# Biomarkers and biometric measures of adherence to use of ARV-based vaginal rings

**DOI:** 10.7448/IAS.19.1.20746

**Published:** 2016-05-02

**Authors:** Randy M Stalter, Thomas R Moench, Kathleen M MacQueen, Elizabeth E Tolley, Derek H Owen, for the Consortium for Ring Adherence

**Affiliations:** 1 Contraceptive Technology Innovation Department, FHI 360 Durham, NC, USA; 2 ReProtect, Inc. Baltimore, MD, USA; 3 Global Health Research Department, FHI 360 Durham, NC, USA

**Keywords:** antiretrovirals, HIV, prevention, adherence, vaginal rings, biomarkers, biometrics, clinical trials

## Abstract

**Introduction:**

Poor adherence to product use has been observed in recent trials of antiretroviral (ARV)-based oral and vaginal gel HIV prevention products, resulting in an inability to determine product efficacy. The delivery of microbicides through vaginal rings is widely perceived as a way to achieve better adherence but vaginal rings do not eliminate the adherence challenges exhibited in clinical trials. Improved objective measures of adherence are needed as new ARV-based vaginal ring products enter the clinical trial stage.

**Methods:**

To identify technologies that have potential future application for vaginal ring adherence measurement, a comprehensive literature search was conducted that covered a number of biomedical and public health databases, including PubMed, Embase, POPLINE and the Web of Science. Published patents and patent applications were also searched. Technical experts were also consulted to gather more information and help evaluate identified technologies. Approaches were evaluated as to feasibility of development and clinical trial implementation, cost and technical strength.

**Results:**

Numerous approaches were identified through our landscape analysis and classified as either point measures or cumulative measures of vaginal ring adherence. Point measurements are those that give a measure of adherence at a particular point in time. Cumulative measures attempt to measure ring adherence over a period of time.

**Discussion:**

Approaches that require modifications to an existing ring product are at a significant disadvantage, as this will likely introduce additional regulatory barriers to the development process and increase manufacturing costs. From the point of view of clinical trial implementation, desirable attributes would be high acceptance by trial participants, and little or no additional time or training requirements on the part of participants or clinic staff. We have identified four promising approaches as being high priority for further development based on the following measurements: intracellular drug levels, drug levels in hair, the accumulation of a vaginal analyte that diffuses into the ring, and the depletion of an intrinsic ring constituent.

**Conclusions:**

While some approaches show significant promise over others, it is recommended that a strategy of using complementary biometric and behavioural approaches be adopted to best understand participants’ adherence to ARV-based ring products in clinical trials.

## Introduction

The importance of adherence to antiretroviral (ARV)-based HIV prevention products has been underscored by results of recent HIV pre-exposure prophylaxis (PrEP) and microbicide studies. In the VOICE and FEM-PrEP trials, poor adherence to vaginal (VOICE) and oral (VOICE and FEM-PrEP) dosing regimens resulted in an inability to determine efficacy [1,2]. Results from the FACTS 001 trial showed 1% tenofovir (TFV) gel was not significantly effective at preventing HIV overall, with poor usage being a major issue [3].

More recently, the need for better adherence support and measures of adherence was demonstrated in trials for ARV-based vaginal ring products. Results of two Phase III clinical trials assessing the safety and efficacy of a one-month vaginal ring containing the antiretroviral drug dapivirine (DPV) for reducing HIV transmission among African women (A Study to Prevent Infection with a Ring for Extended Use [ASPIRE] and The Ring Study) indicated that the product had a moderate effect on preventing HIV transmission overall. However, the effectiveness of the product was higher among sub-groups of women known to have higher levels of adherence [4,5].

Addressing the adherence-related challenges presented in these studies is critical as many other ARV-based vaginal rings are progressing through the development pipeline. Phase I trials of vaginal rings delivering tenofovir disoproxil fumarate (TDF) [6], TFV, maraviroc (MVC) and a combination MVC/DPV ring product [7] have been completed. In addition to ARV-only rings, numerous multi-purpose prevention technology (MPT) vaginal rings that deliver ARVs in addition to contraceptive hormones [8] or drugs that prevent other sexually transmitted infections [9] are in various stages of development. It is anticipated that, over the next few years, multiple additional ring products will move from preclinical to clinical testing of safety and pharmacokinetics (PK) during use ranging from 60 days to 12 months.

Vaginal rings are widely perceived as a way to achieve better adherence, in part because this technology is more convenient and requires less action from the user [10]. Nevertheless, they do not eliminate the adherence challenges exhibited in clinical trials. The results of the ASPIRE trial and Ring Study show that even convenient, easy-to-use products with little requirement for repeated action by the end user may not be used consistently due to side effects, fear of side effects, concerns about partner awareness and disapproval, limited motivation, or other factors. Direct observation of enacted adherence, i.e. observation of the actual act of using the product over time, is impractical for vaginal rings, which can be removed and reinserted at will [10].

Given current development activity, it is important to identify new and better ways of supporting and measuring adherence to these products. The need for objective measures of adherence has been recognized for some time in the microbicide trials field [11,12]. While the use of biometric approaches has been documented for past oral and vaginal microbicide products [13], an exhaustive review of existing and potential biometric methods of vaginal ring adherence has yet to be published. We conducted a landscape analysis of technologies that have potential future application for vaginal ring adherence measurement, including those used for indications outside of HIV prevention, to identify new and improved approaches that could be applied to ring trials specifically. We also evaluate each approach on its respective technical strength and feasibility.

## Methods

As part of the landscape analysis, we conducted a comprehensive literature search, which covered various biomedical and public health databases, including PubMed, Embase, POPLINE and the Web of Science. Published patents and patent applications were searched using the online Patsnap platform (London, UK) and included patent applications filed with the United States Patent and Trademark Office (USPTO) and the World Intellectual Property Organization (WIPO). Once search results were compiled, two reviewers (RMS, DHO) reviewed titles and chose a subset of publications and patent applications for abstract and subsequent full text review.

Abstracts from relevant meetings were also scanned for presentations on biometric approaches to adherence measurement. Conference programs from 2012 to 2015 from meetings of the American Association of Pharmaceutical Scientists, the American Congress of Obstetricians and Gynecologists, the Controlled Release Society, Conference of HIV Research for Prevention (R4P), International AIDS Society (IAS and International AIDS conferences) and the International Association of Providers of AIDS Care (International Conference on HIV Treatment and Prevention Adherence) were included in this search.

Technical experts working on developing, validating and implementing the approaches we identified were contacted to learn more about the specific technologies. Additional experts in the fields of ARV-based ring formulation and development, clinical trial implementation and biometric adherence measurement were also contacted to help evaluate existing approaches to ring adherence measurement as well as discuss the feasibility of adopting new technologies in clinical trial settings.

Each promising approach that was identified through the landscape analysis was evaluated using several criteria, including impact on development and manufacturing, feasibility of implementation in clinical trials, technical strength and cost. Based on how well they met these criteria, and taking into account additional key advantages and disadvantages, methods were designated high, medium, or low priority for further development for use in clinical trials involving ARV-based vaginal ring products.

## Results

We categorized approaches identified through our landscape analysis as either point measures or cumulative measures of vaginal ring adherence (Table 1). Point measurements are those that give a measure of adherence at a particular point in time. Some of the approaches that fall into this category give an immediate evaluation of adherence, which provides the opportunity for real-time intervention with non-adherent participants. Multiple point measurements may be extrapolated to estimate a woman's adherence to the ring over a longer period of time. Cumulative measurements, as we define them, are approaches that measure total use of the product from insertion until the product is returned. Cumulative approaches can either measure ring adherence over a period of time by taking continuous real-time measurements of ring use or by measuring, after the ring has been returned to the study staff, a parameter correlated with the length of time the ring has been used. The former is preferable as it allows for determination of when the product has been removed and for how long.

**Table 1:** Key considerations for development and implementation of point and cumulative measures

	Development/manufacturing	Feasibility of implementation in clinical trials			Technical strength			Cost	
					
	No modification to existing ring required	No major accept- ability issues expected	Minimal clinic staff training required^a^	Clinic staff time/effort minimal^a^	Applicable to active and placebo rings	Allows for immediate feedback on adherence	Other key technical advantages (↑)/limitations (↓)	Estimated cost per ring/assay^c^	Priority level
**Point measures**									
Intracellular drug levels	•	•	•	TBD^b^	–	–	↑ Indicative of longer term use^d^	++	High
Plasma drug levels	•	•	•	TBD^b^	–	–		++	Medium
Vaginal fluid drug levels (swabs, CVL)	•	–	•	TBD^b^	–	–		++	Medium
Saliva drug levels	•	•	•	TBD^b^	–	–		++	Medium
Wise Case	•	–	–	•	•	–	↓ Adherence to device required	+++	Medium
Integrated magnet	–	–	–	–	•	•	↓ Unannounced visits required	+	Low
Integrated RFID tag	–	–	–	–	•	•	↓ Unannounced visits required	+	Low
Breath test	–	–	–	–	•	–		+ +	Low
**Cumulative measures**									
Hair drug levels	•	–	•	•	–	–	↑ Indicative of longer term use	+ +	High
Diffusion analyte in	•	•	•	•	•	–		TBD^e^	High
Diffusion excipient out	–	•	•	•	•	–		TBD^e^	High
Residual drug in ring	•	•	•	•	–	–		+ +	Medium
Colour changes to ring	•	•	•	•	•	•	↓ High variability	+	Medium
Biofilm accumulation	•	•	–	–	•	–	↓ High variability ↓ Biofilm easily removed	+	Low
Integrated sensor	–	–	–	–	•	–		+ ++	Low

a In addition to that required for existing routine clinical trial data/sample collection and analysis activities.

b Depends on whether spot checks are required or if sample collection at scheduled follow-up visits is sufficient.

c In addition to the current cost of ring manufacture; +< 10 USD, ++ 10–100 USD, +++> 100 USD; costs are estimates based on literature and discussions with key experts.

d Only applicable for drugs with intracellular metabolite half-lives substantially greater than half-lives of parent drug in plasma (e.g. TFV).

e Contingent on the analyte/excipient chosen.

•, technology fulfils criterion; –, technology does not fulfil criterion. CVL: cervicovaginal lavage; RFID: radio-frequency identification; TBD: to be determined.

## Point measures

### Integrated radio-frequency identification (RFID) tags or magnets

Spot checks of ring use have the advantage of allowing immediate adherence feedback and support. However, having to remove the ring may be considered cumbersome or intrusive by some participants. Integrating a technology into the ring that could be detected from outside of the body is a possible means to circumvent these limitations. RFID tags and magnets are two examples of such technologies that could be explored for this purpose. Magnets have numerous applications in healthcare, including in magnetic resonance imaging (MRI), hearing aids and ventricular assist devices. RFID tags are increasingly used to track shipments through supply chains, manage supply inventories and validate that certain actions have taken place, such as the administration of the correct drug regimens to patients [14]. An RFID tag or magnet integrated into the ring could be detected externally by portable handheld RFID readers and magnetometers (or Gauss meters), respectively, thus disclosing whether the ring is in place. Ideally, spot checks using RFID or magnetic tags would be conducted during random home visits to reduce the chances of women “outwitting” the test by inserting the ring shortly before a visit.

### Blood drug levels

#### Plasma/serum drug levels

Drug levels in the blood serum and plasma have been used in a number of HIV microbicide and PrEP clinical trials to objectively assess adherence to products and evaluate the accuracy of self-reported measures [15–17]. One limitation of these assays is that certain ARVs have been shown to have short plasma elimination half-lives and can therefore only be used to evaluate short-term adherence for these drugs. TFV and emtricitabine (FTC) have plasma terminal elimination half-lives of 17 hours and 10 hours, respectively, and therefore do not significantly accumulate in plasma over time [18]. Therefore, a detectable drug level is only indicative of recent use. The mean plasma terminal elimination half-life for DPV was shown to be 67 hours [19]. However, it is possible that the ring could be inserted shortly before a clinic visit and show relatively high plasma levels, which could lead to inaccurate assessments of adherence. PK data from users of the DPV ring showed that drug is detectable in plasma at the earliest scheduled time point of 1.5 hours with a gradual increase thereafter [19]. Another concern is the intra- and inter-individual variation of plasma PK profiles that has been observed with ARV use [20], which can make it difficult to draw relative conclusions about good adherence versus poor adherence at a point in time [21].

#### Intracellular blood drug levels

The measurement of accumulated drug metabolites within blood cells has been explored as a more accurate appraisal of long-term ARV adherence. In the iPrEX trial, levels of intracellular tenofovir diphosphate (TFV-DP) and emtricitabine triphosphate (FTC-TP) were measured in peripheral blood mononuclear cells (PBMCs) of individuals taking emtricitabine/tenofovir (FTC/TDF) for PrEP [22]. The FEM-PrEP trial measured TFV-DP levels in upper layer packed cells (ULPCs), which consist of platelets, lymphocytes, monocytes and granulocytes, in addition to evaluating TFV levels in plasma [17]. Given that intracellular phosphorylated metabolites can have longer half-lives, certain microbicides can accumulate in cells over time. For example, intracellular PBMC concentrations are indicative of dosing over the preceding two to four weeks [23,24]. PBMC isolation from blood, however, is rather complex, costly and difficult to conduct in rural study sites. The evaluation of ULPC drug levels may be more feasible as they are more easily isolated from whole blood, and ULPC drug levels have been shown to correlate with PBMC levels with some discrimination between intermittent versus consistent dosing [25]. ULPC drug levels were used in the FEM-PrEP trial with plasma drug levels to construct composite adherence scores for participants, which may provide a more comprehensive picture of participant adherence [17].

An alternative to these methods is the use of dry blood spots (DBS) to assess intracellular ARV levels. While analysis of DBS samples can be just as burdensome and costly as other blood assays, the collection of these samples can be more convenient than standard blood draws, because smaller volumes of blood are needed and can be collected with a simple finger prick. Metabolites of some ARVs have been shown to have longer half-lives in red blood cells, which are abundant in DBS, than in PBMCs. For example, the half-life of TFV-DP has been shown to be approximately 17 days in red blood cells as compared to four days in PBMCs. With DBS TFV testing, it is also possible to detect both the parent drug and intracellular phosphorylated drug metabolites, allowing for the simultaneous testing of recent and longer term drug exposure for drugs that have long intracellular metabolite half-lives [26]. This could potentially reduce assay costs and allow for the estimation of the percentage of participants that engage in “white coat” adherence, instances in which medications are taken shortly before a clinic visit. Comparisons of DBS with other adherence measures have shown that TFV-DP levels in DBS correlated with PBMC drug levels and were negatively correlated with the number of days between monthly pharmacy refills [27].

While the metabolites of some ARVs are known to be detectable intracellularly (e.g. TFV and FTC), the potential of drug assays on cellular populations for use with other ARVs being used in novel ring products remains to be determined. Also, a more complete understanding of the individual variation in plasma and intracellular levels, and the pharmacokinetics of active pharmaceutical ingredients (APIs) is needed including, for example, how quickly the plasma or intracellular levels of a given microbicide agent rise after ring insertion. Without such information, the insertion of vaginal rings worn only for clinic visits might give the impression of adherence. If so, blood draws at random home checks would be required for accurate assessment of adherence.

### Vaginal fluid samples

Vaginal swabs [28], lavage [28–30] and test strips [19] have been used to detect the API in the vagina in previous microbicide trials. This method has an advantage over the previously described point measures that use integrated magnets or electronics because no physical alteration to the ring is needed and API levels are measured directly. However, given that vaginal drug levels can be detected shortly after insertion, it may be difficult to determine whether the ring had been inserted just prior to the visit or at an earlier time. A pharmacokinetic assessment of the DPV ring, for example, demonstrated that drug levels were present in the vaginal fluid at the earliest measurement 1.5 hours after insertion [19]. Another disadvantage is the intrusive nature of the test. This may be alleviated by requesting participants to self-swab under a privacy gown during random at-home checks; a similar approach was successfully used for the self-insertion of vaginal applicators in a previous study [31]. Also, given that vaginal cleansing is widespread in areas of Sub-Saharan Africa, both for regular hygiene and as a pericoital practice, a better understanding of how intravaginal cleansing and product insertion affects vaginal fluid samples is needed [32–35].

### Saliva drug levels

A potential alternative to analysing blood and vaginal fluid samples is the measurement of saliva drug levels to monitor ARV adherence. Saliva drug measurements would allow for sample collection that is easy, fast and non-invasive. Also, saliva samples could be collected in the presence of trial or clinic staff, reducing the possibility of sample manipulation or contamination [36]. Additionally, sample processing time is generally shorter for saliva than for blood samples. To date, much of the research around this approach has been in the context of monitoring ARV drug levels for HIV treatment [37–39]. A few studies have explored this method for use with PrEP regimens as well [40,41]. While high-performance liquid chromatography (HPLC) has been most commonly used for saliva sample analysis, multiple groups have explored thin layer chromatography (TLC) as a low-cost alternative to monitor nevirapine levels in saliva, with mixed results [37,42,43]. As with plasma drug levels, the use of saliva drug levels may not be an ideal method to use alone, given the shorter half-lives of drug relative to intracellular drug levels.

### Wise Case

An indirect approach to estimating adherence to vaginal ring products is to record when and for how long the product is stored outside of the body. This would entail the use of a storage container for the ring whenever it has been removed, a device that would include integrated electronics that can record when the case is opened. This event would then be relayed to a central monitoring server. The concept of the Wise Case is an adaptation of a similar technology, the Wisebag, which has been used to measure use of microbicide gel applicators and has been shown to be a better surrogate of adherence than self-reporting [44,45]. Other similar examples are the Wise Pill container [46] and medication event monitoring systems (MEMS) [47], used for the assessment of adherence to oral medications. With perfect use, this method would provide an extremely accurate measure of ring non-adherence. However, this approach merely transfers the measure of adherence to ring use to the measure of adherence to a method of storage. Therefore, if the ring is removed and not placed in the Wise Case, assessments about adherence based on Wise Case data would be inaccurate. Also, until extra functionality is given to the device so that it can detect if the object being placed inside (e.g. a weight sensor), there is no way of knowing whether the ring was actually placed in or removed from the device at an opening event.

### Volatile taggants

The use of a breath test to detect the presence of chemical taggants added to microbicidal gels has been explored. The taggants, 2-pentyl acetate and 2-butyl acetate, were detectable using a mini-gas chromatograph [48]. In another study, isopropyl butyrate and 2-pentyl butyrate were tried as gel taggants, but were undetectable with a breath test [49]. This method could potentially be adapted to evaluate adherence to the use of vaginal rings. However, the potentially unacceptable taste experienced by some participants in the microbicide studies may reduce acceptability. Also, the reliance on the sampling of breath in general could be problematic, as this may be burdensome to some users. This method requires further study with attention to possible confounding factors, such as the influence of food and alcohol intake, that could potentially result in false positive or false negative results.

## Cumulative measures

### Biofilm accumulation

The measurement of biofilm, a collection of proteins, polysaccharides, DNA and cells that form on the ring when it is inside the vagina, could potentially provide an overall assessment of use. One study that observed biofilm formation on vaginal rings in human participants found not only an increase in the volume of cells accumulated on rings over time, but also a sequential nature of cell adherence, suggesting the need for a layer of epithelial cells before bacteria can readily adhere [50]. A similar sequence of epithelial cell and bacteria accumulation has been observed in studies of non-human primates [51,52]. Therefore, the characterization of biofilm formation on vaginal rings could give an indication of the total length of time worn. Potential limitations include inter-individual variation in vaginal microbiota between participants (perhaps due to the presence of pathogens), which could lead to variations in the rate or type of biofilm accumulation that occurs. Additionally, and probably most critically, the removal, manipulation, or washing of the ring by the participant could lead to biofilm removal or a change in the biofilm's appearance.

### Drug detection in hair

Quantification of drug levels in hair as a measure of drug exposure and adherence has been explored with numerous ARV APIs. Hair samples are easy to collect, store and transport, and the drug remains stable within hair strands for long periods of time making this approach appealing as an adherence monitoring tool. In addition, strong correlations have been established between TFV and FTC levels in hair and other adherence measures in oral dosing studies, including plasma drug levels, PBMC drug levels, MEMS caps records [53] and DBS drug levels [54]. A moderate correlation was demonstrated with self-reported adherence to oral ARV pills [53]. Linear correlations have also been established between oral doses of TFV and hair concentrations, demonstrating an ability to discriminate between various levels of drug exposure [55]. While the assays used for detecting drug in hair samples are costly (i.e. liquid chromatography-tandem mass spectroscopy [LC/MS-MS]), efforts are being made to develop lower-cost assays, such as TLC, to analyse the hair samples [56].

While sample collection is painless and non-intrusive compared to other methods, participant acceptability has been shown to vary across settings and populations. Studies in Kenya and Uganda have reported ≥95% acceptability of the method [53,57], while qualitative data from South Africa shows participants may be reluctant to donate hair due to fear of the hair being used in witchcraft or other means to inflict harm [58]. Also, while only 10–20 strands of hair are required for analysis, repeated sampling may be less acceptable to women. As such, acceptability will likely be context-specific and will need to be understood before data collection takes place in a particular setting. Clearly communicating to participants how the hair samples will be stored and used may help to assuage concerns.

It also should be noted that most studies assessing drug uptake in hair have been done with orally administered drugs. The assessment of drug uptake with vaginal drug delivery is needed. Additional variables that could affect sample integrity should also be investigated further. For example, several studies in rodents have shown that the incorporation of non-ARV drugs into hair is greater in pigmented hair versus non-pigmented hair [59–61]. Also, in many settings, it is quite common for women to treat their hair with chemical products. Studies have shown that bleaching and even washing hair can reduce the uptake of certain drugs [62,63]. Finally, a study of hair drug levels in participants of a PrEP trial in Kenya and Uganda showed that reporting the recent consumption of khat or marijuana was associated with reduced FTC concentrations in hair [53].

### Integrated sensors

Incorporating a component into the ring, such as an indicator strip or electronic sensor, that can detect, transmit, or record biometric data indicative of use could determine how long the ring was present inside the vagina, or even pinpoint specifically when the ring was removed and/or reinserted by the participant.

#### Temperature

Numerous sensors that record vaginal temperature have been described in the literature, including the Neotrend^®^ sensors (Diametrics Medical, Inc, St. Paul, MN), which have been used to observe changes in intravaginal temperatures [64], and the OvuSense sensor that was developed for the detection of ovulation and increased fertility as an alternative to ultrasound [65]. The OvuSense device is the only one of the two that allows for wireless data collection, but its rather large size may not make it practical for use in a vaginal ring. Researchers at Queen's University Belfast have achieved proof-of-concept for an electronic temperature-logging device integrated into silicone vaginal rings. The device was able to accurately measure and record intravaginal temperatures in cynomolgus macaques, with the ability to accurately detect when the ring was removed or inserted [66].

#### pH

Measurement of pH, either with the aid of an integrated electronic component, such as a pH meter, pH detecting microchip, or a simple test strip incorporated into the ring is also a viable biometric marker of use. Normally, the pH in the vagina is between 3.8 and 4.5. However, pH can be highly variable between women and, given the high prevalence of bacterial vaginosis among women in Africa, women's intravaginal pH can reach 6 or higher. If a women's pH is close to neutral (pH = 7), this would make it difficult to differentiate between periods of use and non-use. Having a device that only measures pH when in the presence of a fluid (and not simply when exposed to air) may overcome this barrier and warrants further investigation.

#### Vaginal pressure

The shape and size of vaginal rings are designed to use pressure from the vaginal walls to keep the ring in place after insertion. Using a method that detects pressure placed on the ring could be another way of measuring the amount of time the ring is in place in the vagina. Numerous intravaginal pressure sensors have been developed to measure intra-abdominal pressure (IAP) [67], including wireless models [68], with feasibility testing in human participants suggesting that the sensor was comfortable and easy to insert and had excellent correlation with pressure exerted by participants through various activities [69]. However, this technology has not yet been applied to a vaginal ring.

#### Air exposure

An alternative to using physiologic conditions for the detection of vaginal ring use is to determine the absence of use by measurement of exposure of the ring to air. One such method would be the detection of oxygen or carbon dioxide due to the exposure of the ring to the internal versus external environment. A number of electronic sensors have been used intravaginally to measure oxygen and carbon dioxide levels, including the Neotrend (mentioned above) and Paratrend sensors [70,71]. A simpler, yet potentially less sensitive approach would be to incorporate a substance or test strip into the ring that would become oxidized with exposure to air. Depending on the type of detection mechanism, levels of oxidation could be measured quantitatively or through simple visual inspection, which could then be correlated with an amount of time the ring spent outside the body.

### Diffusion of analytes into or out of the ring

#### Residual drug in the ring

Residual drug levels remaining in ring products after use has been explored as an indicator of use in the recently completed DPV ring trials. Results from a parallel group trial with ring removal time points at 1, 2, 4, 8 and 12 weeks showed that residual levels of DPV in the ring decreased with longer durations of ring use [72]. This approach has been applied to products for other indications as well. Data on residual drugs extracted from explanted contraceptive implants (Sino-Implant (II) and Jadelle) at various time points have been used to estimate monthly and daily levonorgestrel release rates [73]. This methodology could potentially be applied to vaginal rings such that predictive models are applied to residual drug levels in removed vaginal rings at various times to evaluate adherence.

A potential shortcoming of this method is that if only a small fraction of the loaded drug is released, the precision in detecting the small differences in the amount of the drug remaining may be limited. Also, the rate of release of the drug may vary among individuals due to differences in metabolism and vaginal environment as well as behavioural factors such as removals or reinsertions that occur outside the clinic, the environment in which these events occur, how the ring is handled or stored and the use of other vaginal products.

#### Depletion of a non-drug intrinsic or added analyte from the ring

Quantification of the depletion of a non-drug intrinsic ring analyte or of an added inert excipient that has diffused out of the ring during vaginal wear could also be used to estimate adherence. Examples of intrinsic non-drug analytes would include (depending on the ring polymer) catalysts, non-polymerized oligomeric or cyclic silicones or other oligomers or other monomers, and plasticizers. For the added-excipient strategy, potential excipients would ideally be generally recognized as safe (GRAS) materials that would not require additional toxicological testing. However, even if GRAS materials are used, additional API release and pharmacokinetic studies would still be required before clinical testing.

#### Uptake of a vaginal analyte into the ring

The measurement of a material present in the vagina entering the ring is another approach and may be more desirable as it would not require modifications to the ring formulation. However, it would require the identification of a substance that exists in relatively equal concentrations between individuals and would readily diffuse into the ring over time. The concentration of this material would ideally vary little over the reproductive cycle or in the presence of pathogens. Potential substances for this method include cholesterol, physiological ions (e.g. Ca^2 +^, Mg^2 +^, Na^+^, K^+^, Cl^−^), urea and glycerol. Compared to adding an excipient to the ring, this approach would be advantageous from a regulatory standpoint and may be more easily implemented. It will be evident, however, that the diffusion of a particular analyte will differ between types of material used in the ring (e.g. silicone, ethylene-vinyl acetate, or polyurethane), ring sizes, or other factors. Therefore, assays to detect analytes may not be transferrable across ring types or, at the very least, would need re-validation.

### Colour changes to the ring

Observing changes in the colour of the ring has been explored in the Phase III trials of the DPV ring. Indications are that the DPV ring discolours when used over extended time (A Nel, personal communication, 2015). However, no validated scale correlating ring colour to total time worn has been developed. Doing so would be a challenge, given that ring colour changes gradually over time and inter-individual variations in colour changes are likely. For example, women may menstruate while wearing the ring, which could differentially change ring colour relative to non-menstruating women. Therefore, observing ring colour alone will likely have limited ability to quantitatively indicate wear time. However, if visual inspection of the ring was conducted at follow-up clinic visits or even by peers at participants’ homes, it could provide an opportunity to cost effectively provide immediate feedback on adherence and motivate use during unannounced home visits or follow-up clinic visits.

## Discussion

Given findings from recent microbicide and PrEP trials in which a lack of product use resulted in an inability to determine product effectiveness, it is clear that more accurate biometric measures of adherence are urgently needed to ensure that efficacy trials of new ARV-based rings are successful. When assessing potential methods for integration into trials, feasibility of development, manufacturing and implementation, cost, and key technical strengths and disadvantages will ultimately determine which technologies are worthwhile pursuits for future development.

Approaches that require modifications to an existing ring product are at a significant disadvantage, as this will likely introduce additional regulatory barriers and costs to the development process. This is especially true of those approaches that require integrated electronics, which will increase development and purchase costs, as well as extend the development timeline. The temperature-logging ring prototype developed by the University of Belfast, for example, costs approximately €250 per unit [66]. While the general expectation may be that electronics can be made cheaply, the reality is that this is only true if they are produced at large quantities (i.e. millions of units). Clinical trials, however, are unlikely ever to create the need for this kind of volume.

Modifying the ring may also complicate the manufacturing process. The standard regulatory requirement is that Phase III trials use the exact version of the product that is to be offered for commercialization if approved and departures from this principle are not traditionally granted. In this case, additional steps or troubleshooting on the part of the manufacturer would be required, and even if feasible, would drive up costs. Therefore, for measuring adherence in an experimental vaginal ring product, technologies that require simple modifications or no modifications to the ring are preferred. Some of the methods mentioned that require modification, particularly those that can accurately provide real-time measurements of use, could potentially be applied to placebo rings in Phase III trials, however, or as a “gold standard” for comparison in a feasibility study of another biometric or behavioural approach or a vaginal ring dosing study.

It is important for any approach that is implemented in the field to be acceptable to study participants. Approaches that involve no or minor modifications to the ring will likely be more acceptable. Additionally, methods that require little input from the user are preferable as they will be less prone to error. Acceptability considerations go beyond the technology to include whether the way the technology is implemented may adversely affect the trust between trial participants and research staff. Some of the point measures are the researchers’ explicitly expressed distrust about the honesty of the participants’ statements regarding whether they are actively using the ring. Examples include being scanned with a portable RFID reader or magnetometer and use of a breath test for volatile taggants. Approaches that include random home visits raise ethical concerns about maintaining confidentiality and protecting privacy. This may be especially important for young women living with parents or other family members. Random home visits would likely require disclosure of study participation and ring use to other household members, which could increase experiences of disapproval or social harm for some women, and make recruitment and retention more difficult for trial implementers. Some methods (e.g. the collection of hair samples and blood) may be difficult in some settings depending on customs and beliefs on how the samples could be used for harm. Further acceptability research is needed to better understand these issues among populations that will participate in future trials. Unintended effects on the research relationship would need to be carefully considered before using any new approach.

Ideal approaches would also require minimal training, time and effort by the clinic staff, beyond what is currently required in microbicide and PrEP trials. Technologies that use data or sample collection procedures already integrated into prior clinical trials (e.g. blood draws or swabs) would best meet these criteria as clinic staff would already be trained to collect these types of samples. The use of new electronic devices, on the other hand, would require additional training, time and costs. Also, should these devices malfunction or break down in the field, staff must be available to respond and provide technical support. Despite this, cumulative measures could potentially save time for staff, as data collection would take place after the rings are returned and regular spot checks and sample collection would not be needed.

Based on our evaluation, approaches were designated as low, medium, or high priority for further development and introduction into clinical trials. Four approaches have been designated as high priority: intracellular drug levels (particularly DBS), drug levels in hair, the depletion of a non-drug analyte from the ring and the uptake of a vaginal analyte into the ring. While the first two approaches are drug-dependent and are not applicable to placebo rings, the ability of these methods to distinguish between short-term and long-term use (depending on the API) is a significant advantage over plasma drug levels. More research on the systemic absorption of the various ARVs being used in new vaginal rings (e.g. MVC and DPV) and the subsequent incorporation into hair and blood cells is needed, however. Measuring the depletion of a non-drug analyte from the ring or the uptake of a vaginal analyte into the ring would have the added advantage over residual drug measurements of being applicable to both active and placebo rings.

Methods that allow adherence to be measured for both active and placebo rings are advantageous because the information could potentially be used in real or near real time during the trial without compromising study blinding. This could allow adherence-reinforcement activities to be directed to those study participants most in need of it and in time to improve the power of the trial. Second, we believe that an insufficiently recognized benefit is that data on both active and placebo ring adherence could allow comparisons of infection rates across strata of comparable adherence in the two arms. This may be critical in order to make inferences as to the true efficacy of the product. Adherence might plausibly vary *inversely* with risk-taking behaviours, with highly adherent participants also practicing other lower risk behaviours. Thus, without data on placebo adherence to provide the ability to compare the infection rates of similarly adherent participants across arms, it would not be possible to attribute low infection rates in highly adherent participants to the efficacy of the product. We argue that the methods applicable to both active and placebo rings should receive the highest priority for development and testing.

We have assumed perfect adherence to ARV-based rings (i.e. consistent use for the entire period of prescribed use) as the goal during clinical trials when assessing the technologies presented in this paper. However, rings may only need to be in use at or soon before exposure and less than perfect adherence could be sufficient for the product to be effective, both in trials and during use after product approval. Additional pharmacokinetic testing is needed, however, to determine what minimal level of use is needed to achieve steady-state effective drug levels or how far in advance of an exposure event the ring would need to be inserted.

While some approaches show more promise in measuring adherence, no one perfect solution currently exists to objectively measure ring adherence. At least for the time being, method combination, or triangulation, is likely the best approach. This approach includes a combination of biometric approaches (e.g. combining a method to determine longer term adherence such as hair drug concentrations with the integrated magnet approach so that instant feedback on adherence can be provided to participants during the course of the trial) and a combination of biometric measures with composite behavioural measures, such as indexes or scales, to better develop an overall picture of participant adherence [74,75]. When psychometrically validated, scales may better assess the multiple factors likely to contribute to ring non-adherence, so that targeted adherence counselling support can be provided. Furthermore, a validated scale that could be administered prior to randomization and that assesses either the propensity for risk-taking or for adherence, could complement biomarker data (when applicable to both placebo and active products) to determine the true efficacy of the experimental product. Given that new ring products under development include not only ARV-specific but MPT ring products that include drugs for multiple indications, efforts should be made to develop biometric measures that are applicable to a wide range of ring products, not just those for HIV prevention. With many new vaginal ring products currently in development, innovative strategies and technologies must be explored to ensure that product adherence can be properly supported and measured.

## Conclusions

Our review identified numerous approaches that are currently being applied to adherence measurement in clinical trials of ARV-based vaginal ring products or could potentially be applied to such studies in the future. Based on our evaluation of feasibility of development and implementation as well as the technical strength of each approach, we identified several promising methods that warrant further investigation for use in trials. We believe that continuous methods that do not require changes in manufacture of the rings, and can be applied to both active and placebo rings, should receive the highest priority. Some approaches show significant promise over others, but we recommend the adoption of a strategy of method combination, involving the use of complementary biometric and behavioural approaches, to best understand participants’ adherence to ARV-based ring products in clinical trials.
